# Accuracy Assessment of Digital Surface Models Based on WorldView-2 and ADS80 Stereo Remote Sensing Data

**DOI:** 10.3390/s120506347

**Published:** 2012-05-11

**Authors:** Martina L. Hobi, Christian Ginzler

**Affiliations:** 1 WSL Swiss Federal Institute of Forest, Snow and Landscape Research, Zuercherstrasse 111, CH-8903 Birmensdorf, Switzerland; E-Mail: christian.ginzler@wsl.ch; 2 Forest Ecology, Institute of Terrestrial Ecosystems, Department of Environmental Systems Science, ETH Zurich, CH-8092 Zurich, Switzerland

**Keywords:** DSM, DEM, WV2, satellite, aerial images, sensor, CHM, photogrammetry, forest

## Abstract

Digital surface models (DSMs) are widely used in forest science to model the forest canopy. Stereo pairs of very high resolution satellite and digital aerial images are relatively new and their absolute accuracy for DSM generation is largely unknown. For an assessment of these input data two DSMs based on a WorldView-2 stereo pair and a ADS80 DSM were generated with photogrammetric instruments. Rational polynomial coefficients (RPCs) are defining the orientation of the WorldView-2 satellite images, which can be enhanced with ground control points (GCPs). Thus two WorldView-2 DSMs were distinguished: a WorldView-2 RPCs-only DSM and a WorldView-2 GCP-enhanced RPCs DSM. The accuracy of the three DSMs was estimated with GPS measurements, manual stereo-measurements, and airborne laser scanning data (ALS). With GCP-enhanced RPCs the WorldView-2 image orientation could be optimised to a root mean square error (RMSE) of 0.56 m in planimetry and 0.32 m in height. This improvement in orientation allowed for a vertical median error of −0.24 m for the WorldView-2 GCP-enhanced RPCs DSM in flat terrain. Overall, the DSM based on ADS80 images showed the highest accuracy of the three models with a median error of 0.08 m over bare ground. As the accuracy of a DSM varies with land cover three classes were distinguished: herb and grass, forests, and artificial areas. The study suggested the ADS80 DSM to best model actual surface height in all three land cover classes, with median errors <1.1 m. The WorldView-2 GCP-enhanced RPCs model achieved good accuracy, too, with median errors of −0.43 m for the herb and grass vegetation and −0.26 m for artificial areas. Forested areas emerged as the most difficult land cover type for height modelling; still, with median errors of −1.85 m for the WorldView-2 GCP-enhanced RPCs model and −1.12 m for the ADS80 model, the input data sets evaluated here are quite promising for forest canopy modelling.

## 1. Introduction

Digital surface models (DSMs) depict the elevation of surfaces visible from the sensor, such as building tops, tree tops, or unoccluded bare ground [1]. Today, specialists from a large range of disciplines are making use of such models. For example, in forest science DSMs are used to model the canopy surface of forests and analyse its vertical structure [2,3]. Thus, DSMs enable the 3D modelling of the forest canopy, which allows assessments of tree cover [4], estimation of crown structure [5], measurements of canopy heights [6,7] and the detection of canopy gaps [8], including the monitoring of these properties over time. For all the mentioned applications it is crucial to know the accuracy of the input data for the DSM generation as they influence the usability and reliability of the generated results.

In general the preferred data source option for digital surface modelling is a balance between the desired accuracy of the DSM, the costs involved in its creation and the availability of the input data [9]. Remotely sensed data are suitable for DSM generation [10–12] and can be acquired on different platforms (e.g., satellite, airplane) [13]. There are two main types of remote sensing: active systems such as laser or radar, and passive systems such as optical images. In the last two decades airborne laser scanning (ALS) has taken an upturn due to its operability [14]. In forest research airborne laser scanning is often the method of choice, because in forested areas the laser can penetrate to the ground [15]. Airborne laser scanning is costly, however, which limits repeated measurements for the monitoring of changes in the forest. In contrast, passive systems as aerial and satellite images are routinely acquired by national mapping agencies in a continuous cycle, which makes them highly suitable for monitoring over time. Aerial images have to be captured by an airplane, which involves planning the flight and acquiring the permits for data acquisition. A DSM based on RC 30 frame camera images has already been used for an assessment of the increase and decrease of forest area in a mire biotope [16]. Space-borne images provide a cost-efficient alternative to aerial images and can be obtained regardless of various national over-flight restrictions. The launch of IKONOS in 1999 as the world's first commercial sub-meter satellite opened up new possibilities in 3D data capturing. IKONOS can acquire two images of the same region with a ground resolution of approximately 1 m, which allows for the precise extraction of 3D features. The performance of IKONOS for DSM generation was for example evaluated by Baltsavias *et al.* [17] and Eisenbeiss *et al.* [18]. Accuracy studies for DSM generations based on other commercial stereo satellites such as QuickBird in 2001 with a ground resolution of 0.65 m (e.g., [19,20]), WorldView-1 in 2007 with a ground resolution of 0.5 m (e.g., [21,22]), GeoEye-1 in 2008 with a ground resolution of 0.5 m (e.g., [23,24]) and WorldView-2 in 2009 with a ground resolution of 0.5 m (e.g., [25]) followed these developments. So far most of these studies have been presented at conferences, thus accuracy evaluations of DSM derived from the stereo satellite images mentioned above are still ongoing.

Because these very high resolution satellites (VHRS) have a submetric ground resolution, they potentially offer an efficient alternative to airborne surveys for DSM generation. Thus, the question of the input data source to be used now depends on factors other than ground resolution alone. Therefore, the accuracy assessment of the DSM is crucial in the generation process of a DSM [26], as any elevation errors propagate to the final product and can lead to false conclusions, e.g., about forest canopy properties. The accuracy of a DSM depends on a number of variables such as the roughness of the terrain surface, the interpolation function, interpolation methods and three key attributes (accuracy, density, and distribution) of the source data [27,28]. Thus the target land cover type is expected to influence the error budget of the derived DSM [29]. In order to take into account the influence of topographic variations, land cover classes should be distinguished in the accuracy assessment [30,31]. Prior studies showed that the accuracy of a DSM over forested areas is lower than over bare land (e.g., [31]).

For space-based DSM accuracy assessment to be reliable, it is important to know how accurately the satellite image material is georeferenced with the delivered rational polynomial coefficients (RPCs). These coefficients describe the image position by means of two third-order polynomials as a function of the ground coordinates [32]. However, the geopositioning accuracy achieved solely from the RPCs delivered with high resolution satellite imagery is limited [33]. By adding ground control points, the scene orientation can be optimised, but it is of interest for the user to know the accuracy of a DSM without ground control points, because measuring GCPs is time-consuming and in some remote areas not feasible.

In this study, we focus on the evaluation of DSMs generated with photogrammetric methods by using stereo pairs of airborne ADS80 and spaceborne WorldView-2 images. The data sets are one of the best commercially available images at present and provide potentially valuable high-resolution input data for DSM generation. The Leica Geosystem Airborne Digital Sensor ADS80 was released in 2008 and can deliver stereo co-registered, equal resolution imagery in panchromatic, visible and infrared-bands. The Digital Globe's WorldView-2 satellite is operational since 2010 and provides stereo imagery with a panchromatic ground resolution of 0.5 m. As reference for the evaluation of the DSMs ground check point data, stereo-measurements and airborne laser scanning data were used.

Hence, the specific aims of this study are:

1. To assess the accuracy of photogrammetric digital surface models based on airborne ADS80 and spaceborne WorldView-2 stereo images
2. To test the influence of bias-corrected rational polynomial coefficients (RPCs) on the accuracy of the WorldView-2 DSMs
3. To evaluate differences in the accuracy of the derived models based on different land cover types (grass and herb vegetation, forested areas and artificial areas)
4. To discuss the potential of the DSMs for forest applications

## 2. Material and Methods

### 2.1. Study Area

**Figure 1.**
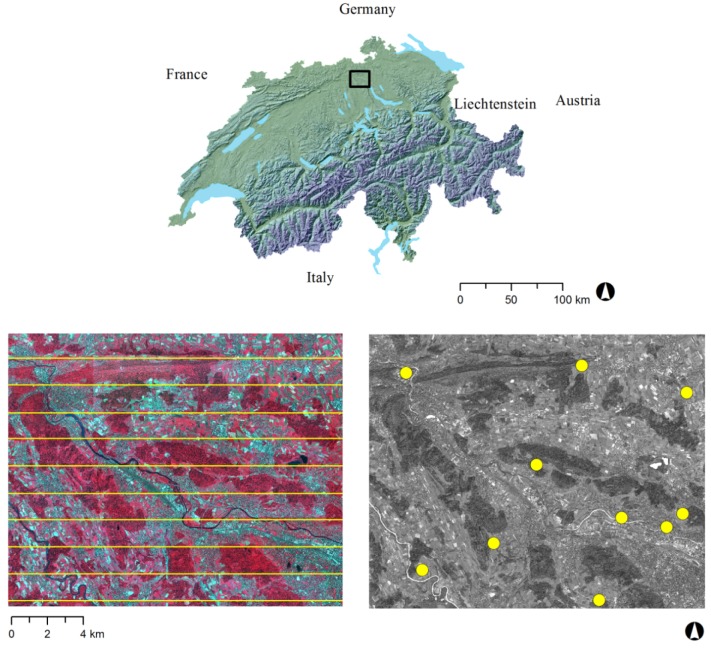
Location of the study area in Switzerland between the cities of Zurich and Baden (top, black rectangle). ADS80 CIR image with 11 centrelines of the stripes with 50% overlap in yellow (bottom left) and WorldView-2 scene with the 10 ground control points (GCPs) for orientation enhancing in yellow (bottom right).

The study area is situated in the eastern part of Switzerland between the two cities of Zurich and Baden (centre image coordinates: 47°25′N and 8°23′E) (Figure 1). The site covers an area of almost 300 km^2^, with a ground elevation ranging from 339 m to 866 m and is characterised by a hilly topography. A small-scale mixture of different land cover classes (urban, rural and forest) characterises this area, which is typical for the Swiss plateau.

### 2.2. Data for DSM Generation

Two optical stereo data sets captured in 2010 were used, one acquired by a spaceborne and one by an airborne digital sensor. These data sets formed the basis for the calculation of three different digital surface models (DSMs).

#### 2.2.1. WorldView-2 Stereo Satellite Images

WorldView-2, operational since January 2010, is the first very high-resolution 8-band multispectral commercial satellite providing a ground resolution of 0.5 m panchromatic and 1.84 m multispectral. The sensor is able to collect stereo images by looking forward and backward from its actual position [34]. The simultaneous acquisition of along-track stereo data has an advantage in terms of reducing radiometric variation because the two images are taken on the same pass. The two images were acquired on 14 July 2010 and show almost no cloud cover. Detailed specifications of WorldView-2 are presented in Table 1.

To describe the object-to-image space transformation of the satellite images, rational polynomial coefficients (RPCs) originating from satellite ephemeris and star tracker observation are delivered with the images. This geometric relation is expressed by 80 coefficients [35]. As these RPCs are generated without ground data, their accuracy is limited [24,33]. To improve image orientation, ground control points (GCPs) measured with the Control Point Editor of SocetSet 5.6 (BAE Systems) were used. Ten well-defined positions visible in the image and the terrain were measured by a GPS (Trimble Geoexplorer XH 2005) with an accuracy of ±10 cm (standard deviation) after differential correction.

#### 2.2.2. ADS80 Stereo Aerial Images

In June and July 2010 stripes of stereo images were recorded with the line scanning camera system Leica Geosystems ADS80. This airborne digital sensor allows the acquisition of panchromatic, colour and near-infrared images at the same time. For our analysis 11 stripes of the CIR (coloured near-infrared) image data were used to cover the area with a ground resolution of 25 cm. The image acquisition and the aerial triangulation were carried out by the Swiss Federal Office of Topography (swisstopo), and the residuals of the orientation were reported with ±1 pixel. Details on the ADS80 digital sensor can be found in Table 1.

### 2.3. Reference Data

To evaluate the accuracy of the calculated DSMs, ground check point data, stereo-measurements and airborne laser scanning data were used. As the accuracy of a DSM also depends on the land cover type [29], for the stereo-measurements and the laser scanning data three land cover classes were distinguished: herb and grass vegetation, forested areas and artificial areas.

### 2.3.1. Ground Check Points

Thirty-six check points distributed over the study area were measured in a ground survey in the summer of 2011. Three-dimensional positions of these points were determined with a sub-decimetre GPS with differential correction (Leica GPS1200) to get superior accuracy. The height accuracy of the 36 ground check points was ±0.02 m (standard deviation). Each of these points was situated on flat terrain and was used as reference for the generated surface models. The z-coordinate of the check points could be compared directly to elevation above sea level in the three DSMs at the x- and y-coordinates of the check points, as they were situated on sport fields or park grass where only minor vegetation changes could be expected.

#### 2.3.2. Stereo-Measurements

In the Swiss National Forest Inventory (NFI) continuous landscape variables are interpreted on the same ADS80 stereo images as those used in our study. In the NFI, landscape variables are measured on a sampling grid (0.5 km × 0.5 km) using interpretation plots of 50 m × 50 m (for details see [36,37]). Within each interpretation plot, the variables are measured on 25 equally spaced (10 m) lattice points arranged in a point design. A photo interpreter assigns each lattice point to a thematic surface cover class using a 3D softcopy station (Stereoanalyst Leica) [38]. In addition to the surface cover, each lattice point is assigned the photogrammetrically measured elevation information (m a.s.l.). For our study, the thematic surface cover classes were aggregated to three land cover classes, *i.e.*, herb and grass vegetation (NFI class 11), forested areas (NFI classes 1-4) and artificial areas (NFI classes 21-22). Repeated measurements in the framework of the Swiss NFI (n = 25,250) showed a median difference of 0.08 m with a normalized median absolute deviation of 1.21 m. In the context of the stereo-measurements we thus talk about agreement and not about accuracy.

#### 2.3.3. Airborne Laser Scanning (ALS)

Airborne laser data from swisstopo were acquired using a TerraPoints ALTMS 2536 [39]. The ALS data in the study area have a mean density of 1.5 points per m^2^. The height accuracy all over Switzerland is reported with ±0.50 cm (standard deviation) [40], but an accuracy analysis in comparison with the ground check points in the target area showed a height accuracy better than ±0.10 cm (standard deviation). The data were acquired in the years 2001 (April), 2002 (February–May) and 2003 (March–June). For the accuracy assessment the ALS data were assigned to three land cover types derived from the Swiss land use statistics from the period 2004–2009 [41].This survey is based on a network of sample points at a distance of 100 m × 100 m and distinguishes six principal land cover domains: artificial areas, grass and herb vegetation, shrubby vegetation, tree vegetation, bare land and water bodies. For our study, the two classes of shrubby vegetation and tree vegetation were aggregated into the single category of forested areas. A total of 28,611 windows of 10 m × 10 m were drawn around each sample point of the grid. Within these windows maximum values of the different DSMs were derived, which allowed for a robust comparison of the values. To avoid a bias the sample was limited to windows where all the pixels could be matched successfully. This resulted in at least 1,300 evaluated windows per land cover type. To calculate the differences between the DSMs, the maximum of the ALS data point was subtracted from the maximum values of the target DSMs.

### 2.4. DSM Generation

A 3D softcopy station (SocetSet 5.6, BAE Systems) and a commercial GIS software (ESRI, Arc Map 10) were used for the processing of the image data and the generation of three different DSMs (WorldView-2 RPCs-only DSM, WorldView-2 GCP-enhanced RPCs DSM and ADS80 DSM). The DSMs were created based on the stereo image data with the Next-Generation Automatic Terrain Extraction (NGATE) of SocetSet 5.6 [42,43]. The NGATE uses both image correlation and edge matching to generate a DSM whereby every pixel is matched many times [44]. The strategy is based on an image correlation window size of 13 × 13 pixels. During seven iterations it goes through different minification levels starting with 64 and ending at level 1. The DSMs were calculated with a grid size of 1 m; for visual analysis hillshades were created.

Based on the satellite data two different DSMs were generated, one using the orientation with the delivered RPCs and tie points only, and one where ground control points were used to enhance image orientation. Tie points were collected for both models during the triangulation to refine the mathematical relationship between ground and image space. To check the image orientation with the RPCs supplied and the GCPs used, the root mean square errors (RMSE) of the x-, y- and z-coordinates of triangulation were calculated.

The orientation of the aerial images was computed by swisstopo using GPS/IMU and aerial triangulation with GCPs. As the ADS80 aerial image data consists of 11 overlapping (50%) stripes, the DSMs were calculated separately for each stripe. For the mosaicing the most nadir part (close to the centreline of the stripe) of the DSM of each stripe was used.

**Figure 2.**
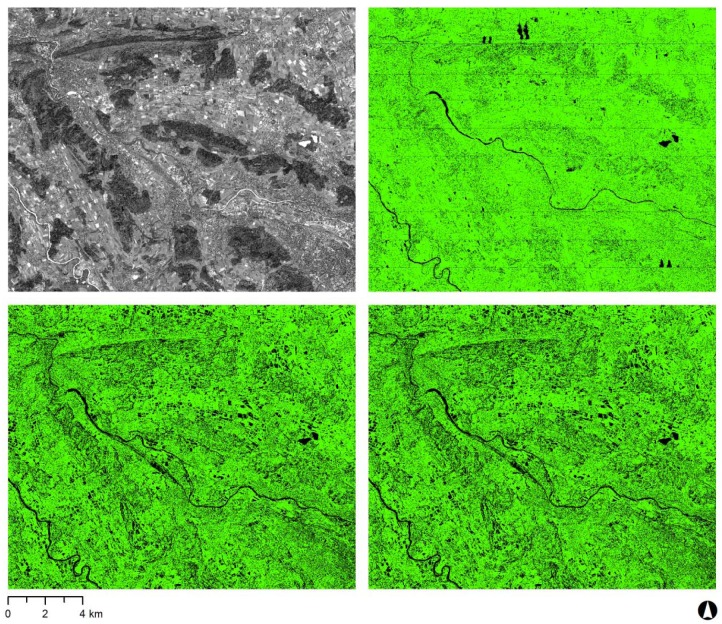
WorldView-2 Pan scene (top left) and figure of merit (FOM) maps of the three DSMs (WorldView-2 RPCs-only DSM, WorldView-2 GCP-enhanced RPCs DSM and ADS80 DSM). Pixels displayed in green were matched successfully and incorporated in the subsequent analysis. Pixels displayed in black are interpolated only and were masked out. FOM map of ADS80 DSM (top right; 87.45% matched cells and 12.55% interpolated cells), FOM map of WorldView-2 RPCs-only DSM (bottom left; 71.16% matched cells and 28.84% interpolated cells) and FOM map of WorldView-2 GCP-enhanced RPCs DSM (bottom right; 71.43% matched cells and 28.57% interpolated cells).

A numerical value called the figure of merit (FOM) was assigned [45] by the terrain extraction process in SocetSet. FOM is ranging from 0 to 100 which shows for each pixel how successful the image matching process was. Only raster cells with FOM ≥ 32 were used for the subsequent analysis, as all cells with FOM < 32 were interpolated during the matching process (Figure 2). This ensured that featureless areas such as lakes, rivers, clouds and cloud shadows were masked out, as image matching is highly limited in these areas.

### 2.5. Accuracy Assessment

The assessment of the accuracy of the digital surface models is crucial for all further calculations and collection of 3D feature data. The three reference data sets (GPS measurements, stereo-measurements, and airborne laser scanning) were used for this evaluation.

#### 2.5.1. Error Distribution

**Figure 3.**
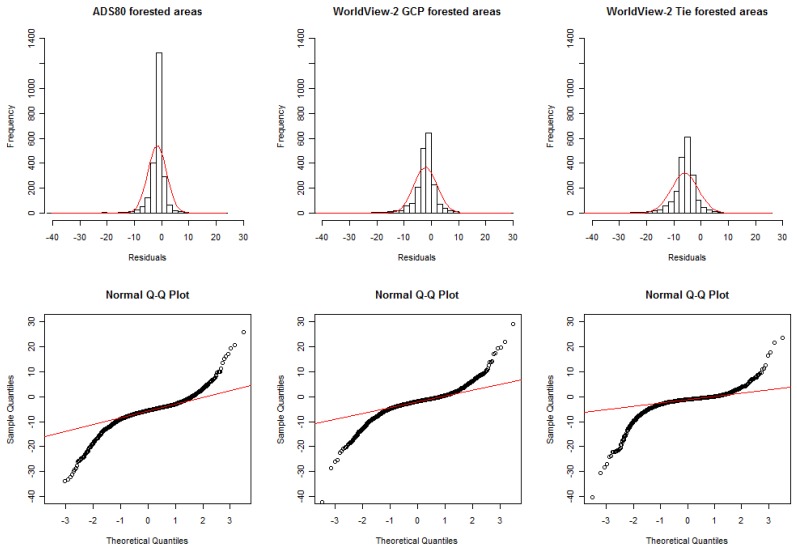
Non-normal error distribution from the comparison of the three DSMs with stereo-measurements in the forested areas. Histograms with superimposed normal distribution and normal Q-Q plots.

DSM errors are usually not normally distributed [29], major and minor outliers being very common. Thus it is important to visualize the error distribution using a histogram with a superimposed curve indicating the normal distribution [26]. As a second step, the so-called quantile-quantile plot can help to check for deviations from the normal distribution (Figure 3). Clearly, the errors are not normally distributed, and thus robust accuracy measures had to be used. Regarding the histogram with the red line indicating the normal distribution, the deviations are evident from the sharper peak around the mean and the longer tails due to a larger number of negative outliers. In the quantile-quantile (Q-Q) plot, the deviations are visible due to the sigmoid shape of the error distributions compared to the straight line of the normal distribution.

#### 2.5.2. Accuracy and Measures

The accuracy of the DSMs was characterised by four robust statistical measures that were tested for their suitability for non-normal error distributions by Höhle and Höhle [26]. The median, the normalized median absolute deviation (NMAD) and the 68.3% and 95% sample quantiles were calculated with the open source statistical software R [46]. Being more resilient to outliers in the dataset, NMAD was used as a measure of the standard deviation:

(1) $$\text{NMAD}=1.4826\cdot {\text{median}}_{\text{j}}(\left|\Delta {\text{h}}_{\text{j}}-{\text{m}}_{\Delta \text{h}}\right|)$$

where Δh_j_ denotes the individual errors j = 1,…,n and m_Δh_ is the median of the errors. For comparison with other studies, root mean square errors (RMSE) were also calculated. When calculating RMSE, a threshold for eliminating outliers was applied [47]: an error was classified as an outlier if |Δh_j_| > 3*RMSE.

## 3. Results and Discussion

### 3.1. Digital Surface Models

**Figure 4.**
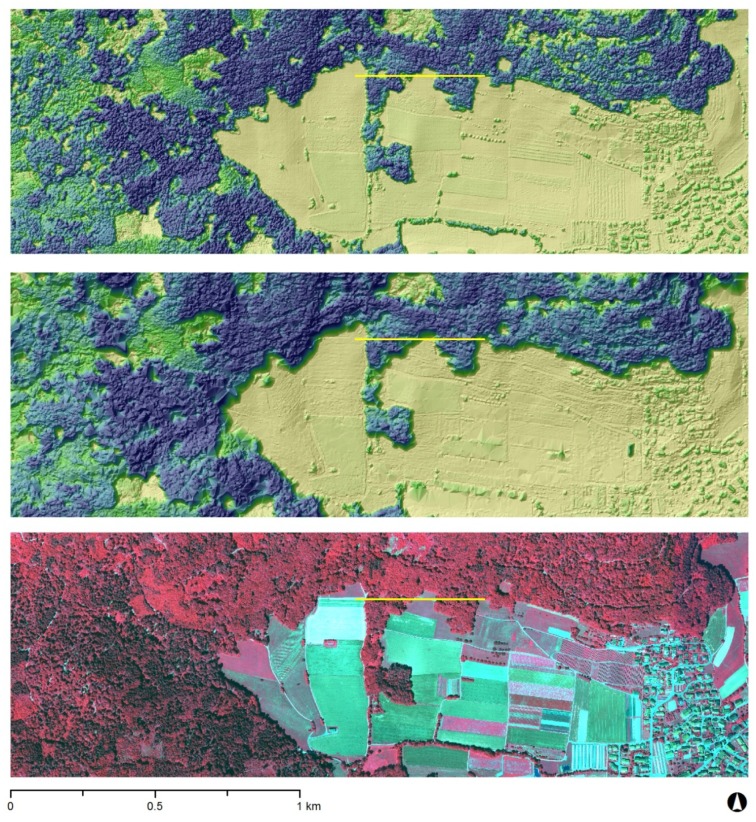
Hillshades of the ADS80 DSM (top) and the WorldView-2 GCP-enhanced RPCs DSM (centre) as well as the ADS80 CIR image of the chosen extent (bottom). Profile of Figure 5 in yellow.

Examples of the WorldView-2 DSM with GCP-enhanced RPCs and the ADS80 DSM are given in Figure 4. The ADS80 DSM looks somewhat smoother and is visually more precise as small objects such as single trees, hedges and houses can be resolved. In general the differences between both surface models are rather small, however it seems that there are more artefacts caused by matching uncertainty on the WorldView-2 GCP-enhanced RPCs DSM.

**Figure 5.**
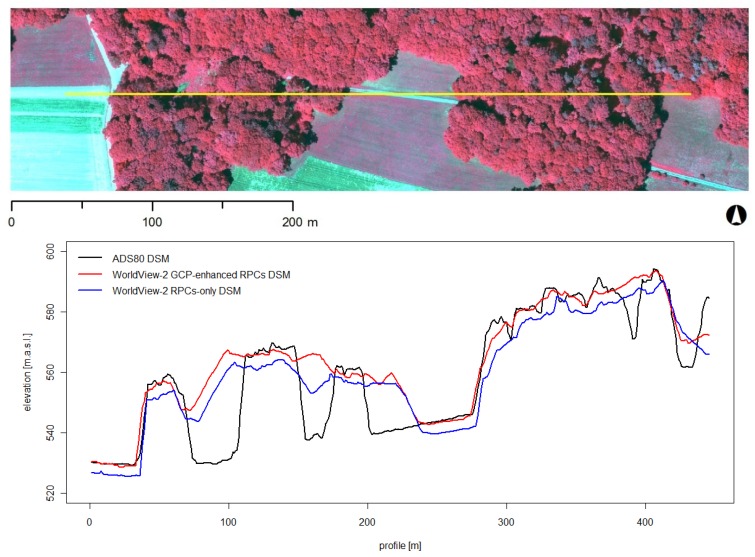
Profile through an exemplary forest stand of the study area to show the difference between the DSM based on airborne (ADS80) and spaceborne (WorldView-2 RPCs-only DSM and WorldView-2 GCP-enhanced RPCs DSM) stereo image data.

An example of the differences between the DSMs based on airborne (ADS80) *vs*. spaceborne (WorldView-2) stereo images is given in Figure 5, using a profile through a forest stand of the study area (*cf.* Figure 4). It shows that the WorldView-2 GCP-enhanced RPCs DSM represents the whole picture in good quality but the ADS80 DSM is able to retrieve more details and finer-scale variations of the forest canopy. The profile curve of the WorldView-2 RPCs-only DSM generally lies below the profile of the two other DSMs. Larger gaps in the canopy are mapped by all three DSMs, however the ADS80 DSM is also able to model smaller gaps in the canopy.

### 3.2. WorldView-2 Image Orientation with RPCs

The root mean square errors (RMSE) derived from the triangulation report showed a good accuracy of the 10 GCPs used (Table 2). With the bias-corrected RPCs the satellite images showed an orientation with a mean horizontal accuracy of 0.56 m and a vertical accuracy of 0.32 m.

### 3.3. DSM Accuracies

#### 3.3.1. Ground Check Points

A comparison of the 36 ground check points in flat terrain with the three different DSMs showed a median error <3.7 m and a NMAD <0.5 m for all three models (Table 3). The ADS80 DSM showed the highest accuracy with a median error of 8 cm and a NMAD of 21 cm. The WorldView-2 GCP-enhanced RPCs DSM showed good accuracy with a median error of −24 cm and a NMAD of 22 cm. When comparing the vertical accuracy measures of the two WorldView-2 DSMs, a clear improvement can be recognised when in addition to the tie points GCPs are used. The two World-View-2 DSMs however had a tendency to underestimate the actual surface height on average. Note that the sample size for these comparisons varies due to the fact that interpolated pixels were masked out.

#### 3.3.2. Stereo-Measurements

The descriptive statistics of the error distribution of the DSMs compared to stereo-measurements is shown in Tables 4–6. The ADS80 DSM best represented the reference surface heights in all the three land cover classes.

The refinement of the WorldView-2 model with the GCP-enhanced RPCs was reflected in the vertical agreement measures. The three DSMs were on average suggesting a bias towards an underestimation of actual height. In general the modelling of the forested areas showed the greatest errors.

#### 3.3.3. Airborne Laser Data

A summary of the statistics for the error distribution of the three DSMs compared to airborne laser data by land cover type is provided in Tables 7–9. For artificial areas, the ADS80 DSM best represents the reference surface heights. The WorldView-2 GCP-enhanced RPCs DSM, however, shows on the one hand the smallest median error for the herb and grass vegetation, but has on the other hand a bigger NMAD than the ADS80 model. For forested areas the WorldView-2 GCP-enhanced RPCs DSM and the ADS80 DSM both show similar median errors but with different algebraic signs. The NMAD of the two models is for forested areas of the same order. The improvement of the WorldView-2 DSM with the bias-corrected RPCs is reflected in the vertical accuracy measures. Both WorldView-2 models have a tendency to underestimate actual surface height on average, whereas the ADS80 DSM is in two cases slightly overestimating the reference heights.

## 4. Discussion

### 4.1. WorldView-2 Image Orientation Based on RPCs

The orientation of the satellite imagery produced by the delivered RPCs could be refined with 10 ground control points (Table 3). In a study by Gianinetto [48] based on Cartosat-1 images, the best results of the refinement of image orientation was achieved using at least nine regularly distributed GCPs. The fact that there are discrepancies between the RPCs derived coordinates and the true ones has already been pointed out in other studies, for example by Baltsavias *et al.* [17] using stereo IKONOS data, and by Noguchi *et al.* [49] based on stereo QuickBird images. The orientation of satellite images will only be as accurate as the RPCs, and there is no practical way to improve upon the sensor orientation via purely analytical means. However, with a modest requirement for ground control, bias-corrected RPCs can be obtained, which in the case of IKONOS and QuickBird imagery imply a sub-metre geopositioning skill [32]. Thus, an important question to answer before the calculation of a digital surface model is how accurate the data have to be given certain scientific objectives. The results of our study indicate that without any ground truth data, DSMs based on WorldView-2 stereo images can achieve vertical accuracies with a median error lower than 5.5 m independent of the target land cover type. This accuracy may be good enough for small-scale applications over large areas or where limitations in the accessibility of remote areas make the collection of ground truth data impossible.

### 4.2. Improvement of WorldView-2 DSM with GCP-Enhanced RPCs

Of the three reference data sets, the WorldView-2 DSM with GCP-enhanced RPCs achieved much better vertical accuracies than the WorldView-2 DSM where solely the RPCs were used for image orientation. This resulted in an improvement of the median vertical accuracy of 3.5 m for different land cover types based on the stereo-measurement data set. The importance of bias correction of the RPCs defining the image orientation before DSM calculations was already mentioned by Baltsavias *et al.* [17]. Our study showed that without a correction of the image orientation, the bias in orientation will propagate to a bias in modelled surface height.

### 4.3. Vertical Accuracies in Flat Terrain

The ADS80 DSM emerged in this study as the most accurate model for surface heights in flat terrain. It showed a median error of 8 cm and an NMAD of 21 cm, which can be regarded as very low (Table 3). There are no studies based on ADS80 stereo images with which these values could be compared. Haala *et al.* [15] worked with ADS40 SH52 stereo images, which are comparable with the ADS80 images used in this study. Their generated DSM was compared with ground reference points situated on paved areas such as small roads or parking lots. They used NGATE of SocetSet for their DSM generation, as we did in this study. After gross error elimination (±3*RMS), they achieved very high accuracies with mean errors of −1.1 cm based on images with a GSD of 8 cm, and of 1.9 cm based on images with a GSD of 20 cm. The ADS80 images used in our study have a GSD of 25 cm, and the resulting vertical errors are only slightly larger than those reported by Haala *et al.* [15]. Waser *et al.* [16] generated a DSM based on CIR aerial images RC 30 and compared it to ALS data. The observed z-differences were 80 cm, but it has to be taken into account that their ALS data were acquired under leafless conditions, in contrast to our images taken in summer. It is not clear in the study of Waser *et al.* whether the ALS data or the RC 30 data are more accurate.

The WorldView-2 DSM with GCP-enhanced RPCs performed also well in the accuracy assessment. Vertical accuracies of this DSM on the bare ground of 24 cm (NMAD 22 cm) can be considered as very good for a DSM derived from stereo satellite images (Table 3). Both WorldView-2 DSMs have a tendency to underestimate the reference height of the ground check points. This tendency is also visible in the comparison of these two DSMs with the ALS data and the stereo-measurements. We believe that there still exist some amount of orientation bias for the WorldView-2 GCP-enhanced RPCs DSM which could not have been eliminated within the triangulation process under the usage of the GCPs. This can explain the still remaining discrepancy between the WorldView-2 GCP-enhanced RPCs DSM and the ADS80 DSM.

Accuracy assessment studies based on comparable images like IKONOS data with a ground resolution of 0.82 m achieved vertical accuracies of 60–80 cm [17]. Eisenbeiss *et al.* [18] also achieved a height accuracy of 0.5–1.0 m in open areas for IKONOS with sophisticated matching algorithms. It is known that for QuickBird stereo images with a ground resolution of 0.78 m, a measured average vertical error of 1.38 m can be reduced by linear regression to 0.29 m [19]. This finding coincides with another study of QuickBird images where a linear error with a 68% level of confidence of 1.2 m was reported [50]. Studies with GeoEye-1 stereo images with the same ground resolution of 0.5 m as WorldView-2 reported mean errors of 0.2 m on sporting fields and 0.5 m on bare ground [24]. These studies show that the WorldView-2 sensor evaluated here acquires stereo images that are a solid basis for state-of-the-art digital surface models holding great potential for the modelling of environmental variables not only over large extents but also with high spatial resolution.

### 4.4. Accuracy as a Function of Land Cover Type

The differentiation of three land cover types showed that the accuracy of a digital surface model varies strongly with land cover. In the comparison of the three DSMs with the two reference data sets (stereo-measurements and airborne laser data) herb and grass vegetation emerged as the easiest land cover type to model, whereas forested areas were the most difficult. The ADS80 DSM performed best in these comparisons with the two reference data sets, followed by the WorldView-2 GCP-enhanced RPCs DSM in second place and the WorldView-2 RPCs-only DSM coming in third.

Regarding the agreement assessment based on stereo-measurements, the ADS80 DSM achieved median vertical errors of less than 1.1 m and the WorldView-2 GCP-enhanced RPCs DSM errors of less than 1.9 m in all land cover classes. The modelling of herb and grass vegetation and artificial areas showed similar accuracies with errors even dropping to less than 50 cm for the WorldView-2 GCP-enhanced RPCs DSM and less than 5 cm for the ADS80 DSM. Forested areas produced median errors of less than 1.9 m for the WorldView-2 GCP-enhanced RPCs DSM and even less than 1.1 m for the ADS80 DSM.

In the comparison with the airborne laser data set, the errors per land cover type were somewhat smaller in the herb and grass vegetation as well as in forested areas than in the evaluation with the stereo-measurement data. The WorldView-2 GCP-enhanced RPCs DSM even performed better than the ADS80 DSM for the herb and grass vegetation. Regarding the forested areas the WorldView-2 GCP-enhanced RPCs DSM showed slightly smaller errors than the ADS80 DSM but the deviation of the two models was similar. This however can result from a bias in data acquisition. The airborne laser data underestimate actual surface heights in the herb and grass land cover type due to the fact that vegetation was lower during spring, when the ALS data was acquired, than in summer, when the images were captured. The herb and grass land cover type includes crop and corn which can have a considerable influence. For forested areas the leaf-off acquisition of the ALS data in spring time and the seven to nine years difference in acquisition have to be taken into account. With the methodological approach by only comparing the maximum values of a cluster we attempted to filter the time differences out, but the influence of the fact that the ALS data was acquired in spring time still remains. Most likely these circumstances explain why the ADS80 DSM seems to overestimate the reference surface heights and the WorldView-2 GCP-enhanced RPCs DSM shows smaller errors than the ADS80 DSM. Regarding artificial areas, the ADS80 DSM is again most accurate because there is no bias in vegetation height. In general the matching success is dependent on the similarity of the left and right images used for DSM generation. In forested and urban areas the degree of similarity can be degraded due to small-scale variations in microtopography. These areas show a high complexity in their structure which can lead to situations, where objects visible in one image cannot be detected in the homologous images. The image matching success in these situations is reduced, which influences the accuracy of the digital surface model.

This differentiation of land cover type is crucial for a better understanding of the accuracy of a DSM in general and especially as the focus of application of these study results lies in forest areas. Image matching in forested areas is more susceptible to difficulties associated with low image contrast and shadowing, as shown by an assessment of IKONOS DSMs in comparison with LiDAR data for differing land cover classes [31]. These difficulties resulted in a lower accuracy for forested areas (RMSE 5.3 m) compared to bare ground (RMSE 2.4 m) and various urban areas (RMSE 2.3–4.7 m). Another study using five classes (bare ground, urban, rural, forest, water) to test a DSM from IKONOS with interferometric synthetic aperture radar (InSAR) reference data came to the conclusion that forest is the land cover type with the biggest differences between the models [30]. Nevertheless, with vertical accuracies lower than 5.6 m on average in all land cover classes including forests the evaluated digital surface models are suitable for modelling various vegetation structures.

### 4.5. DSMs for Forest Applications

Accurate information on surface height and 3D structure of a forest landscape is valuable for many applications in forest science. If a digital terrain model is available for the selected area, a canopy height model can be calculated by subtracting the digital terrain model from the digital surface model [3,8,51]. This allows for investigating forest structure over large areas, and it is a method that can be applied in remote areas with limited access and difficulties for field sampling.

Mapping forest canopy height is a valuable tool for estimating stand-level structures such as top height [52] or stem volume [53] and the characterisation of canopy structure [8]. Canopy gap dynamics can be observed and measured extensively in time and space when canopy height models of different time steps are available. Specifically, these canopy height models can be used to map gap size, shape complexity, vegetation height diversity and gap connectivity [54]. In the context of disturbance dynamics, the method is efficient for monitoring insect outbreaks, forest fires or windthrow, and it allows for a fast estimation of the extent of damage to forest stands. Based on airborne laser scanning data, Mathys [55] presented an approach for mapping and quantifying canopy gaps after disturbances. Canopy gaps defined by their extent (area) and their spatial characteristics (outline/area) were plotted on a ‘gap map’ for each forest stand, and were used for a consistent monitoring of the forest for resource management.

In the absence of such disturbances, local, small-scale gap dynamics dominate forest structure. In a canopy height model, small tree groups and medium-sized gaps can be detected easily. Automatic gap recognition was evaluated e.g., by Vepakomma *et al.* [8] based on a airborne laser scanning canopy height model. The algorithm used in their study was able to estimate canopy gaps smaller than 5 m^2^ in boreal forests. In our study, the visual analysis of the ADS80 DSM demonstrated the potential for mapping smaller gaps, whereas the WorldView-2 DSMs showed a limitation to larger gaps. This means that it will not be possible to map individual tree heights, but it has to be evaluated to what extent canopy gaps can still be determined from the canopy height model.

## 5. Conclusions

We were able to show that: (1) using GCPs the RPCs defining the orientation of the WorldView-2 images can be improved; (2) the WorldView-2 DSM with GCP-enhanced RPCs achieves much higher accuracy measures than the WorldView-2 DSM where only the RPCs are used; (3) the accuracy of the WorldView-2 GCP-enhanced RPCs DSM is similar to the ADS80 DSM (2*GSD); (4) the accuracy of a DSM varies with land cover type; and (5) forested areas are the most challenging areas for surface height modelling among the land cover types evaluated here. The image matching algorithm NGATE (BAE system) is state of the art and was applied to one of the best commercially available aerial (ADS80) and satellite (WorldView-2) images. Thus, the results of our study provide a good basis for answering the question of which sensors are suitable for generating accurate digital surface models and provide an update on the current capacity of DSM generation.

The accuracy comparison of the three surface models showed that very high-resolution satellite stereo data are a valuable alternative to aerial stereo data for surface modelling if the delivered RPCs are bias-corrected with GCPs. A digital surface model based on WorldView-2 images can achieve a vertical accuracy lower than 5.6 m (equals 11.2*GSD) on average in all land cover classes, even in remote areas where field measurements are not possible. When using bias-corrected RPCs, this accuracy can be improved to vertical median errors of less than 1.9 m (equals 3.8*GSD) in all land cover classes. DSMs based on ADS80 can achieve vertical median errors to an accuracy lower than 1.2 m (equals 4.8*GSD) on average in all land cover classes.

These accuracies of the WorldView-2 and ADS80 DSMs show the potential for accurate modelling of forest canopy height. Using the presented techniques, it becomes possible to assess the overall structure of the forest canopy. Vertical canopy structure of a forest can be evaluated based on the different developmental stages of forest patches visible in the digital surface model and the detection of canopy gaps. Assessing the forest canopy height however is only feasible, if a terrain model of the same accuracy of the target area is available. Such terrain models representing the bare ground surface are mostly acquired by means of airborne laser scanning, because accurate global terrain models are still lacking. If a terrain model for a target area is once calculated, with photogrammetric methods it becomes economic to produce series of canopy height models of different dates to monitor changes. Updating the third dimension becomes efficient and costly laser flights do not have to be repeated. Up to now the presented method shows its limits when it comes to single-tree analyses. Questions such as which minimum gap size can be detected in the canopy height model and to what degree such gap recognition can be automated still remain the subject of future research.

## Figures and Tables

**Table 1.** Characteristics of the sensors used (WorldView-2 and ADS80).

	**WorldView-2**	**ADS80**
Acquisition date	14 July 2010	24 June, 7 and 16 July 2010
Sensor type	commercial stereo satellite	CCD-line digital aerial camera
Ground sample distance (GSD)	PAN: 0.5 m GSD at nadir	0.25 m GSD
	MS: 1.84 m GSD at nadir	
Altitude	770 km	3 km
Spectral range (used bands in bold)	**Pan: 450–800 nm**	Pan: 465–676 nm
	Coastal: 400–450 nm	Red: 604–664 nm
	Blue: 450–510 nm	Green: 533–587 n
	Green: 510–580 nm	Blue: 420–492 nm
	Yellow: 585–625 nm	**Near-infrared: 833–920 nm**
	Red: 630–690 nm	
	Red Edge: 705–745 nm	
	Near-IR1: 770–895 nm	
	Near-IR2: 860–1040 nm	
Number of scenes used	1	11
Swath width	16.4 km at nadir	3 km
Orientation accuracy	6.5 m circular error at 90% confidence (CE90)	±0.25 cm

**Table 2.** Root mean square errors (RMSE) of the 10 ground control points (GCPs) used for the bias correction of the rational polynomial coefficients (RPCs) of the WorldView-2 satellite images.

	**RMSE X [m]**	**RMSE Y [m]**	**RMSE Z [m]**	**RMSE total [m]**
GCP	0.45	0.66	0.32	0.86

**Table 3.** Robust vertical accuracy measures of the digital surface models based on ground check points in flat terrain.

**Flat Terrain**	WorldView-2 RPCs-only - Ground truth	WorldView-2 GCP-enhanced RPCs - Ground truth	ADS 80 - Ground truth
Sample size	24	26	35
50% quantile (median) [m]	−3.63	−0.24	0.08
NMAD [m]	0.48	0.22	0.21
68.3% quantile [m]	−3.34	−0.02	0.14
95% quantile [m]	−2.88	0.47	0.39
RMSE (without outliers) [m]	(n = 24) 3.58	(n = 25) 0.33	(n = 35) 0.23

**Table 4.** Herb and grass vegetation. Vertical agreement measures of the three digital surface models in comparison with the stereo-measurements.

**Herb and Grass**	**WorldView-2 RPCs-only - stereo data**	**WorldView-2 GCP-enhanced RPCs - stereo data**	**ADS 80 - stereo data**
Sample size	2,384	2,371	2,923
50% quantile (median) [m]	−3.85	−0.43	−0.04
NMAD [m]	0.67	0.57	0.29
68.3% quantile [m]	−3.55	−0.16	0.09
95% quantile [m]	−1.70	1.27	0.91
RMSE (without outliers) [m]	(n = 2,380) 3.92	(n = 2,342) 0.91	(n = 2,889) 0.53

**Table 5.** Forested areas. Vertical agreement measures of the three digital surface models in comparison with the stereo-measurements.

**Forested Areas**	**WorldView-2 RPCs-only - stereo data**	**WorldView-2 GCP-enhanced RPCs - stereo data**	**ADS 80 - stereo data**
Sample size	2,007	1,974	2,355
50% quantile (median) [m]	−5.53	−1.85	−1.12
NMAD [m]	2.69	2.34	1.32
68.3% quantile [m]	−4.38	−0.90	−0.58
95% quantile [m]	0.37	3.54	1.95
RMSE (without outliers) [m]	(n = 1,985) 7.19	(n = 1,939) 3.98	(n = 2,306) 2.63

**Table 6.** Artificial areas. Vertical agreement measures of the three digital surface models in comparison with the stereo-measurements.

**Artificial Areas**	**WorldView-2 RPCs-only - stereo data**	**WorldView-2 GCP-enhanced RPCs - stereo data**	**ADS 80 - stereo data**
Sample size	987	971	1,272
50% quantile (median) [m]	−3.74	−0.26	0.01
NMAD [m]	1.06	0.86	0.46
68.3% quantile [m]	−3.27	0.18	0.23
95% quantile [m]	0.58	3.69	2.99
RMSE (without outliers) [m]	(n = 973) 4.74	(n = 947) 2.06	(n = 1,252) 1.28

**Table 7.** Herb and grass vegetation. Vertical accuracy measures of the three digital surface models in comparison with the airborne laser data.

**Herb and Grass**	**WorldView-2 RPCs-only - ALS data**	**WorldView-2 GCP-enhanced RPCs - ALS data**	**ADS80 - ALS data**
Sample size	4,295	4,332	7,172
50% quantile (median) [m]	−3.49	−0.07	0.24
NMAD [m]	0.72	0.63	0.48
68.3% quantile [m]	−3.15	0.24	0.51
95% quantile [m]	−2.11	1.20	1.45
RMSE (without outliers) [m]	(n = 4,230) 3.98	(n = 4,244) 1.20	(n = 7,072) 0.85

**Table 8.** Forested areas. Vertical accuracy measures of the three digital surface models in comparison with the airborne laser data.

**Forested Areas**	**WorldView-2 RPCs-only - ALS data**	**WorldView-2 GCP-enhanced RPCs - ALS data**	**ADS 80 - ALS data**
Sample size	1,379	1,323	1,342
50% quantile (median) [m]	−4.36	−0.52	0.87
NMAD [m]	3.12	2.88	2.45
68.3% quantile [m]	−3.09	0.52	2.20
95% quantile [m]	0.92	4.68	6.04
RMSE (without outliers) [m]	(n = 1,326) 8.02	(n = 1,256) 5.06	(n = 1,281) 7.06

**Table 9.** Artificial areas. Vertical accuracy measures of the three digital surface models in comparison with the airborne laser data.

**Artificial Areas**	**WorldView-2 RPCs-only - ALS data**	**WorldView-2 GCP-enhanced RPCs - ALS data**	**ADS 80 - ALS data**
Sample size	1,768	1,723	2,248
50% quantile (median) [m]	−4.91	−1.23	−0.07
NMAD [m]	2.47	2.27	1.15
68.3% quantile [m]	−3.81	−0.35	0.29
95% quantile [m]	−1.91	1.38	2.10
RMSE (without outliers) [m]	(n = 1,756) 7.46	(n = 1,692) 4.18	(n = 2,188) 2.82
